# Brain fingerprints along the language hierarchy

**DOI:** 10.3389/fnhum.2022.982905

**Published:** 2022-09-15

**Authors:** Juan Zhang, Liping Zhuang, Jiahao Jiang, Menghan Yang, Shijie Li, Xiangrong Tang, Yingbo Ma, Lanfang Liu, Guosheng Ding

**Affiliations:** ^1^State Key Laboratory of Cognitive Neuroscience and Learning, IDG/McGovern Institute for Brain Research, Beijing Normal University, Beijing, China; ^2^Department of Psychology, School of Arts and Sciences, Beijing Normal University, Zhuhai, China; ^3^School of Psychology, Beijing Normal University, Beijing, China; ^4^State Key Laboratory of Cognitive Neuroscience and Learning, Center for Cognition and Neuroergonomics, Beijing Normal University, Zhuhai, China

**Keywords:** brain fingerprint, functional connectivity, language hierarchy, fMRI, individual identification

## Abstract

Recent studies have shown that the brain functional connectome constitutes a unique fingerprint that allows the identification of individuals from a group. However, what information encoded in the brain that makes us unique remains elusive. Here, we addressed this issue by examining how individual identifiability changed along the language hierarchy. Subjects underwent fMRI scanning during rest and when listening to short stories played backward, scrambled at the sentence level, and played forward. Identification for individuals was performed between two scan sessions for each task as well as between the rest and task sessions. We found that individual identifiability tends to increase along the language hierarchy: the more complex the task is, the better subjects can be distinguished from each other based on their whole-brain functional connectivity profiles. A similar principle is found at the functional network level: compared to the low-order network (the auditory network), the high-order network is more individualized (the frontoparietal network). Moreover, in both cases, the increase in individual identifiability is accompanied by the increase in inter-subject variability of functional connectivities. These findings advance the understanding of the source of brain individualization and have potential implications for developing robust connectivity-based biomarkers.

## Introduction

For a long time, neuroimaging studies on human brains have been primarily concerned with the generic principles of brain function that are shared across people, with relatively little attention paid to inter-subject variability. In the seminal work conducted by Finn et al., individual variability in brain functional organization was found to be both robust and reliable (Finn et al., 2015). It is possible to identify a target subject from a sample database by computing the spatial similarity of the target subject’s brain functional connectivity (FC) profile against the FCs’ of the database ones, similar to a “fingerprint.” Following this work, further studies have detected various brain features that may act as a “fingerprint” (Liu et al., 2018, 2020; Sareen et al., 2021), proposed novel methods to improve the accuracy of individual identification (Amico and Goni, 2018; Cai et al., 2021), and related the brain fingerprinting features to behavioral traits (Kaufmann et al., 2017).

Yet, what makes our brains unique remains poorly understood. Understanding the source of brain individualization is important for several reasons. First, it will provide critical information for improving the accuracy of individual identification. Second, it will help establish the link between individual differences in brain function and individual differences in cognition and behavior, which in turn may have important implications in precision medicine. Finally, it can provide valuable information for evaluating to what extent the group-level results about brain function can be applied to unknown individuals.

Currently, only a few studies have explored factors that potentially influence individual identifiability, including: (i) the temporal window used to compute FC profiles. It has been reported that the greater identifiability occurred at longer time scales (Van De Ville et al., 2021); (ii) the anatomical loci. Across the whole brain, the connectivity profiles of the frontoparietal network and medial frontal networks were most distinctive for individuals (Amico and Goni, 2018); and (iii) factors affecting fMRI data which might be unique to individuals and stable enough across time, including global signals (Chen and Hu, 2018), head motion and brain anatomy (Finn et al., 2015).

The above work has been mainly focused on the physiological or structural aspects of the brain. Few studies have examined the roles of the functional aspects of the brain in individual identification. In particular, are the high-order functions (such as those supporting story comprehensions) or the low-order functions (such as those supporting auditory perception) of the brain more critical for individual identifiability? There are at least two possibilities: on the one hand, an individual’s brain involved in low-order functions may show a high degree of stability across time, therefore facilitating individual identification; on the other hand, brains involved in high-order functions may vary greatly among people, thus making individual discrimination easier.

A further question is, how important are those task-evoked neural processes compared to task-independent intrinsic processes in distinguishing individuals? Several studies have performed individual identifications across resting states and obtained a high accuracy of above 90% (Finn et al., 2015; Horien et al., 2019). In comparison, identifications made across the resting state and a set of tasks typically produced lower accuracies ranging from about 60% to 85% (Kaufmann et al., 2017; Amico and Goni, 2018). Among the many factors (such as the characteristics of head motion and data length) potentially accounting for the differences in identification accuracy, one possibility is that, under resting states, subjects are actually engaged in a set of active mental processes, including unconstrained verbally mediated thoughts, monitoring, and episodic and autobiographical retrieval processes (Binder, 2012). Therefore, the results of identification across resting states might come from a combination of contributions from both state-independent and state-specific neural processes. Instead, the results of rest-task identification may better capture the contribution of state-independent processes to brain individualization. Yet, no study has systematically investigated the contribution of state-independent, low-order and high-order processes to brain fingerprints.

This study addresses the above two questions by tapping into the hierarchical nature of language. In our experiment, each subject underwent a resting-state fMRI scan, then listened to stories played backward, stories scrambled at the sentence level, and stories played forward during fMRI scanning. For each of the three tasks, brain imaging data were acquired from two separate scan sessions. The three tasks were assumed to involve increasingly complex cognitive processes, while the resting state was used to create a baseline condition. For the backward-played speech, which would appear as meaningless audio streams, subjects should be mainly engaged in low-level acoustic analysis. For the sentence-scrambled story, subjects would need to additionally recognize single words and combine words into sentences (termed as “middle-level linguistic/semantic operations”). For the intact story, in addition to the perceptual and linguistic/semantic computations, subjects would need to combine single sentences into coherent mental models that allow for inferences and conceptual associations. We termed these processes recruited specifically by story comprehension as “high-level conceptual processes.”

To investigate the contribution of low-order and high-order functions of the brain to individual identification, we predicted subjects’ identities across two scan sessions corresponding to the same task, and then compared the success rates (SRs) among the three task conditions. The state-independent neural process (the baseline) is assumed to play a role in both resting and task states. To assess its contribution, we conducted identifications between the resting session and each task session. Finally, to understand why individual identifiability may differ along the language hierarchy, we compared the degree of within-subject stability and inter-subject variability of FCs among the four conditions (including the three task conditions and condition of rest-task pairs).

We first performed individual identification based on the whole-brain functional connectomes. To establish a closer relationship between task-evoked brain functions and individual identifiability, we further conducted individual identification using FC profiles of single functional networks. Three networks known to be critically involved in speech processing were investigated, including a primary auditory network, a perisylvian language network and a frontoparietal network (Price, 2010). In addition, the default mode networks (DMN) which have been suggested to be actively engaged in resting states and a set of high-order functions are also examined (Yeshurun et al., 2021).

## Materials and methods

### Subjects

A total of 30 college students (females, aged between 18 to 35 years) who were native Chinese speakers and proficient in English participated in this study. The criterion applied to screen participants included: (i) having passed the Test for English Majors-Band 8; (ii) scoring above 7 on the International English Language Testing System (IELTS); or (iii) scoring above 95 on the Test of English as a Foreign Language (TOEFL). The data of three subjects were excluded from further analyses due to excessive head movements (more than 3 mm or 3 degrees) during one or more sessions of the fMRI scanning. All subjects were right-handed and had no history of neurological, psychiatric or language disorders.

### Experimental procedure

Stimuli for the experiment were generated from a set of cartoon stories (each lasting ∼60 s) told by two female Chinese speakers during fMRI scanning. Each story was told in both Chinese and English. A noise-canceling microphone (FOMRI-III, Optoacoustics Ltd., Or-Yehuda, Israel) was used to record the speech. The recordings were further de-noised offline using Adobe Audition CS6 (Adobe Systems Inc., United States). Three types of audio clips (lasting 60–62 s) were created from those recordings. The first type of audio clip was the raw stories played forward (intact). The second type (sentence-scrambled) was created by randomly shuffling the sentences of the first half of a story and keeping the second half intact. The third type (backward) was created by presenting the first half of a story waveform-reversed in time and keeping the second half intact. For the latter two conditions, the intact parts of the stories were not included in the analyses. A more detailed description of the stimuli presentation is provided in the Supplementary material (Supplementary Figures 1, 2).

**FIGURE 1:**
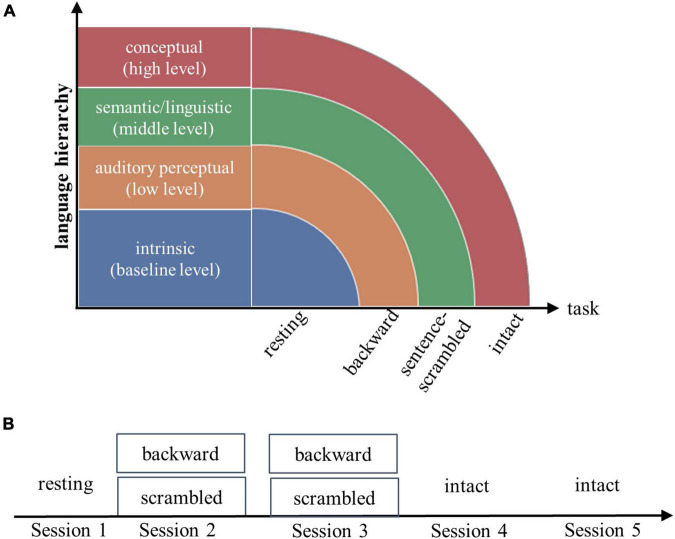
Experimental design **(A)** and fMRI scanning scheme **(B)**. Each subject underwent a resting-state fMRI scanning, then followed by three tasks: listening to short stories presented waveform-reversed in time (backward), stories scrambled at the sentence level, and stories played forward (intact). The resting state and three tasks were assumed to engage increasingly complex processes along the language hierarchy. For each of the three tasks, the brain imaging data were collected from two successive scan sessions.

Each subject underwent five fMRI scan sessions over 2 days. On the first day, following an 8-min resting-state scan session, subjects listened to backward-played stories and sentence-scrambled stories (presented in separate blocks) during two successive scan sessions. On the second day, subjects listened to intact stories during two successive scan sessions (Figure 1). The contents of stories differed between successive sessions.

Half of the subjects were exposed to the Chinese version of audio clips and the other half were exposed to the English version. As bilingualism is not the focus of the current study, we pooled the two subgroups of data together. This study was undertaken with the understanding and written consent of each subject and was approved by the Institutional Reviewer Board of Beijing Normal University.

### MRI acquisition

Imaging data were collected with a 3T Siemens Trio scanner in the MRI Center of the Beijing Normal University in China. For the functional scan, a gradient echo-planar imaging sequence was applied with the following parameters: repetition time = 2,000 ms, echo time = 30 ms, flip angle = 90°, field of view = 220 mm^2^, 33 interleaved slice, voxel size = 3.125 mm^3^ × 3.125 mm^3^ × 4 mm^3^. Additionally, high-resolution T1 structural images were acquired using an MPRAGE sequence. The parameters were: repetition time = 2,530 ms, echo time = 3.39 ms, flip angle = 7°, FOV = 256 mm^2^, and voxel size = 1.0 mm^3^ × 1.0 mm^3^ × 1.33 mm^3^.

### Imaging data preprocessing

The fMRI imaging data were preprocessed using DPARSF (Yan and Zang, 2010),^1^ which integrates the preprocessing modules of Statistical Parametric Mapping (SPM12).^2^ The steps of preprocessing included slice timing adjustment and realignment for head-motion correction, spatial normalization to the Montreal Neurological Institute (MNI) space, resampling into a voxel size of 3 mm^3^ × 3 mm^3^ × 3 mm^3^, and smoothing with an isotropic Gaussian kernel (FWHW = 7 mm). The preprocessed images were further detrended, nuisance variable regressed, and high-pass filtered (1/128 Hz). The nuisance variables included the five principal components of signals in the white matter and cerebrospinal fluid masks (Behzadi et al., 2007) and Friston’s 24 motion parameters (including each of the six motion parameters of the current and preceding volume, plus each of these values squared) (Friston et al., 1996).

### Data analysis

#### Functional connectivity estimation

Identification for individual subjects was made based on their brain FC profiles and performed across two sessions of the same task and across the rest and each of the task sessions. For each task session, the time series corresponding to the task blocks were extracted. Before the data extraction, the time series of each brain subregion were normalized in time using z-score. The data were shifted back in time by 4 s to account for the hemodynamic lag of blood-oxygen-level-dependent (BOLD) signals. As previous studies have shown that data length can influence the accuracy of identification (Van De Ville et al., 2021), we extracted an equal number of time points (*N* = 32) from each session. This number was determined by the maximum data length of the backward and the scrambled conditions.

**FIGURE 2:**
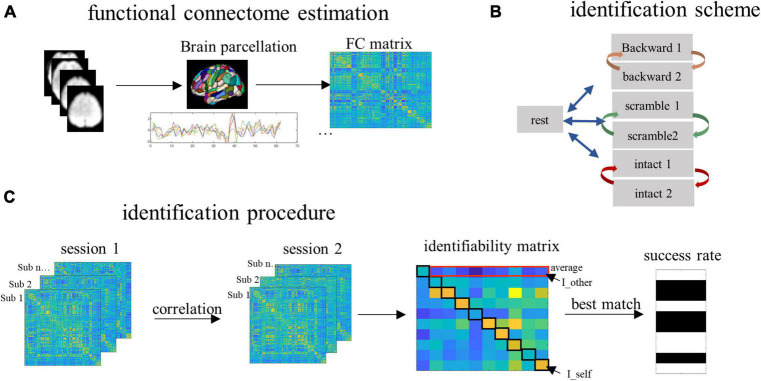
The procedure of data analysis. **(A)** The whole brain was partitioned into 368 parcels. Time series corresponding to the task blocks were extracted and concatenated to compute the functional connectivity (FC). **(B)** To detect the contribution of task-independent brain processes, we paired the resting scan with each of the six task scans for the identification. To detect the contribution of low- and high-order brain processes, for each task condition, we paired the two successive scan sessions corresponding to the same task. **(C)** To predict subjects’ identities, the FCs from a database set were correlated with the FCs from the target set, resulting in an identifiability matrix. Based on this matrix, we obtained the within-subject FC similarity (quantified by the I_self), between-subject FC similarity (quantified by the I_other), individual identifiability (quantified by the I_diff, which is computed by I_self minus I_other), and the group-level success rate of identification.

To estimate FC, we partitioned the whole brain into 368 subregions using the Shen-368 Atlas (Salehi et al., 2020; Luo and Constable, 2022). This was a fine-grained atlas obtained by integrating the parcellation of cortex from Shen et al. (2013), subcortex from the anatomical Yale Brodmann Atlas (Lacadie et al., 2008), and cerebellum from Yeo et al. (2011). Pearson correlation coefficients between each possible pair of subregions were computed, resulting in a 368 by 368 connectivity matrix (Figure 2A). This was done for each subject and each condition, such that each subject had a total of seven connectivity matrices representing connectivity patterns during resting and the three tasks (two matrices for each task).

#### Identification using whole-brain functional connectivities

To detect the contribution of the four types of information to brain individualization, we conducted identification for individuals across time using the pairing scheme illustrated in Figure 2B. Specifically, to detect the task-independent intrinsic processes (the baseline level), identification was conducted across the resting state session and each of the six task sessions. To detect the auditory perceptual processes (the low level), identification was conducted across the two sessions of the backward condition. To detect the linguistic/semantic processes (the middle level), identification was performed across the two sessions of the sentence-scrambled condition. Finally, to detect the conceptual processes (the high level), identification was performed across the two sessions of the intact condition. For each level, the FCs derived from one scanning session served as the database and another session served as the target. The two sessions then changed the roles (Figure 2B).

To predict subjects’ identities, an identifiability matrix was defined as Pearson’s correlations between the database and the target (Amico and Goni, 2018). The main diagonal elements of the matrix represent the FC similarity of the same subjects across sessions, referred to as I_self. The off-diagonal elements of the matrix, averaged by columns, represent the FC similarity of subjects (from the database session) with other subjects (from the target session). This was referred to as I_other. The result of I_self minus I_other was referred to as I_diff, which reflects the identifiability of individual subjects for a given fold of identification (Figure 2C).

In addition to the I_diff which served as a continuous variable to quantify the identifiability of individual subjects, we also calculated the group-level SR of identification. For a given subject, if the I_self was larger than every other element on the same row in the identifiability matrix, the identification was successful, otherwise it failed. In other words, if a subject’s FCs in the database showed greater similarity with his/her own FCs than with any other subjects in the target set, the identification succeeded. This procedure was iterated over all subjects. The accuracy of identification was measured as the percentage of subjects whose identities were correctly predicted out of the total number of subjects in the group (Finn et al., 2015).

We first evaluated the identifiability index (I_diff) and SR for each pair separately and then averaged the results of corresponding pairs into one.

#### Statistical analysis

Non-parametric permutation tests were performed to assess whether the obtained identification accuracy was significantly above chance. In the permutation, the subjects’ identities of the target set were randomly assigned and then the identification was performed. This procedure was repeated 1,000 times to create a null distribution for each session pair. Then for each condition, the null distributions of identification pairs were combined and the maximum SR from the null distributions was extracted as the threshold for the given condition. In addition, Chi-squared tests were applied to compare the SR of identification among the four conditions.

#### Comparison of within- and between-subject similarity

The success of individual identification mainly depends on how similar was the connectome patterns of a given subject with his/her own FCs (quantified by the I_self) and with the FCs of other subjects (quantified by the I_other). To gain insights into the potential differences in individual identification along the language hierarchy, we compared the two variables among the four conditions using paired *t*-tests. Multiple comparisons were corrected using a false discovery rate (FDR) at *Q* = 0.05.

#### Identification based on single functional networks

In the above analyses, the whole-brain connectome may encode not only task-related information, but also multiple task-unrelated neurophysiological processes as well as task-free intrinsic processes. To establish a closer relationship between task-related processes and individual identifiability, we further performed the identification based on the FC profiles of single functional networks. Currently, most brain functional networks reported in the literature are created from resting-state fMRI data. To more accurately detect those functional networks closely involved in the language task, we conducted brain network parcellation using an independent data set involving 61 subjects listening to a 10-min real-life story while undergoing fMRI scanning. Applying a recently-developed technique (Ji et al., 2019; Barnett et al., 2021), a group-mean 368 by 368 FC matrix was clustered into 15 networks. Details of network partition are presented in the Supplementary material. The four networks of interest, including the auditory, language, DMN and frontoparietal networks were selected via visual inspection and validated by comparing with corresponding templates on Neurosynth^3^ in terms of spatial overlap.

#### Tests for the robustness of results

##### Functional connectivities assessed with a different brain atlas

To test the robustness of the major findings, we re-analyzed the data by assessing the functional connectomes using the Schaefer atlas which partitioned the brain into 400 areas (Schaefer et al., 2018). Then we assessed individual identifiability along the language hierarchy based on 400 by 400 FC matrices.

##### Identification using a different strategy of condition pair and longer data length

To validate the main results, we adopted a different strategy of condition pair to assess individual identifiability, which allowed us to use more time points to compute FCs. Given the hierarchical nature of language, it is reasonable to assume that, in addition to the state-independent intrinsic activities, those state-specific processes engaged in the low-level task (e.g., listening to the backward-played story) are also engaged in the high-level task (e.g., listening to the intact story) (as illustrated in Figure 1A). Thus, the degree of similarity of subjects’ FCs between the low- and the high-level tasks should mainly depend on the low-level task, which in turn would largely determine whether a subject can be identified between the conditions. Nevertheless, we noted that while the shared components of low- and high-level tasks can improve the accuracy through shared state-specific contribution, differences between state-specific activities could reduce the performance. In this way, changes in identification accuracy between conditions may underestimate the contribution of the targeted processes (the shared state-specific processes).

Following the above logic, we combined one dataset of the intact condition with the dataset of the rest, the backward, the sentence-scrambled, and the second intact conditions separately. Then identification was conducted between the two datasets for each of the four pairs. We predicted that the identifications of the rest-intact, backward-intact, scrambled-intact and intact-intact conditions should follow a similar pattern as the identification of the rest-task, backward-backward, scrambled-scrambled and intact-intact sessions.

For each condition, a total of 64 time points were extracted to compute FCs. For the backward and sentence-scrambled conditions, this was done by concatenating the corresponding time series from two scan sessions. For the intact condition, this was done by concatenating the corresponding time series from two blocks. For the resting state, the first 64 time points were extracted. Time series were normalized within a session before the concatenation.

## Results

### Individual identifiability along the language hierarchy based on whole-brain functional connectivities

**FIGURE 3:**
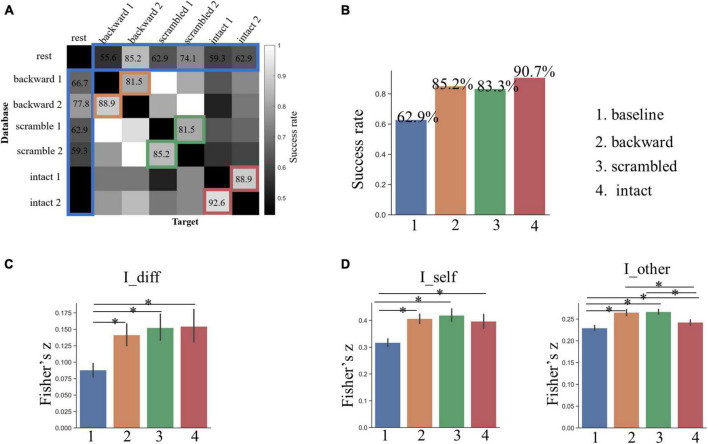
**(A)** The result of individual identification along the language hierarchy based on whole-brain FCs. **(B)** The success rate of identification across all possible pairs of sessions. Grids marked by colors are the interests of this study. The bar plot shows the group-level success rates averaged across corresponding pairs for the four conditions. **(C)** Individual identifiability quantified by I_diff. **(D)** Within- and between-subject similarity across sessions quantified by I_self and I_other, respectively. The asterisk indicates a significant difference between two conditions at *p* < 0.05 after FDR correction. The error bars denote the standard deviation of means.

Based on the whole-brain FC patterns, we predicted subjects’ identities across the rest and each of the six task sessions with a mean accuracy of 62.9% (the baseline, ranging from 44.44 to 85.19% across pairs), which was much higher than the best performance (22.2%) from the permutation test (Figures 3A,B). Across the two sessions for the backward condition, which was presumed to involve low-level acoustic processing, the mean SR of identification was 85.2% (ranging from 81.48 to 88.89%). Across the two sessions for the sentence-scrambled condition, which was presumed to involve additionally middle-level linguistic and semantic processing, the mean SR of identification dropped slightly to 83.3% (ranging from 81.48 to 85.19%). The greatest SR was achieved across the two sessions for the intact condition (mean = 90.74%, ranging from 88.89 to 92.59%), which was presumed to involve further high-level conceptual processing.

For all three task conditions, the SR of individual identification across two sessions was significantly higher than that of the baseline (*p* < 0.005, by Chi-squared test). Nevertheless, the differences in SR among the three task conditions were not statistically significant (*p* > 0.25).

The analysis of the I_diff revealed a similar pattern: the individual identifiability increased along the language hierarchy, and the I_diff for all three tasks was greater than that of the baseline, but had no statistically significant differences among the three tasks (Figure 3C).

### Changes in within- and between-subject similarity along the language hierarchy

To gain insights into why the individual identifiability varied along the language hierarchy, we compared the degree of within- and between-subject similarity (quantified by the I_self and I_other, respectively) in FC patterns across corresponding sessions. The within-subject similarity (or stability) in whole-brain FC profiles was the greatest across the two sessions for the sentence-scrambled condition, next for the backward condition, and the weakest for the intact condition (Figure 3D). This pattern was partially consistent with our prediction that the functional brains involved in the low-order tasks varied less across time than the brains involved in high-order tasks. The FC profiles within subjects were the least stable across the rest-task sessions (the baseline condition), which was significantly lower than that of all three task conditions (*p* < 10^–4^, by paired *t*-test).

The distribution of between-subject similarity resembled that of within-subject similarity. That is, compared to the higher-level intact condition, subjects were more similar to each other in their brain FCs under the lower-level backward and the sentence-scrambled conditions (*p* < 10^–3^) (Figure 3D). This pattern was consistent with our prediction that the functional brain involved in higher-order functions is more variable across people. Still, compared to three task conditions, the brain FCs varied more between subjects across the rest and task states (*p* < 10^–3^).

Together, those results suggest that the greater individual identifiability across the task sessions than that across the rest and task sessions might be related to the more stable brain FCs across the task sessions. The greater individual identifiability under the high-order condition than that under the low-order conditions might be related to the larger between-subject variability.

### Network-based identification

The whole-brain FCs during tasks may be shaped by both task-related and task-unrelated processes, such as intrinsic activities or physiological noises. To gain more insights into the task-related information that potentially makes our brains distinguishable, we performed the identification using the FCs of functional networks.

**FIGURE 4:**
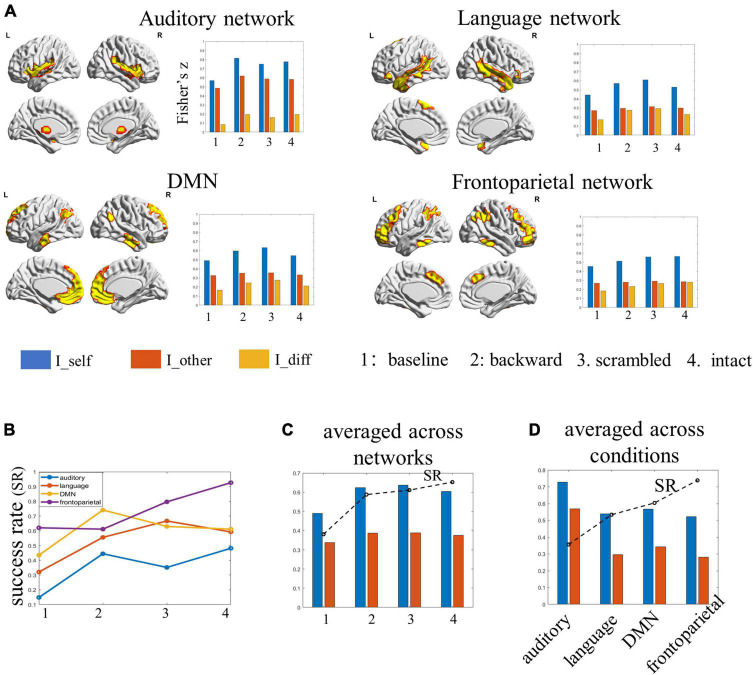
The results of individual identification based on single functional networks. **(A)** The spatial map of the selected functional networks and the individual identifiability, within- and between-subject similarity in the FC profiles of each network. **(B)** The success rate of each network in identifying individuals under each condition. **(C)** The success rate and the within- and between-subject similarity for each condition, averaged by networks. **(D)** The success rate and the within- and between-subject similarities for each network, averaged by conditions.

Among the four examined networks, the auditory network achieved the lowest identification accuracies (ranging between 14.8 and 48.1%) under all four conditions. Further analyses showed the within-subject similarity in network FC profiles was the largest in the auditory network, especially under the backward condition. At the same time, the between-subject similarity in FC profiles was also the largest in this network. In other words, different subjects seemed to have similar FCs in the auditory network across sessions, potentially leading to the low SR in identification. The language network achieved SRs ranging from 32.1 to 59.2%, which also tended to increase along the hierarchy: the performance was lowest for the baseline, better for subjects under the backward condition, and the best for subjects under the sentence-scrambled condition, which then dropped down slightly for subjects under the intact condition. The DMN achieved SR ranging from 43.5 to 74.1%. The best performance obtained by this network was for subjects under the backward condition. The frontoparietal network performed the best (ranging from 61.1 to 92.6%) in distinguishing individuals under most conditions. Consistent with the performance of the language network and the whole-brain connectome, the frontoparietal network also distinguished individuals with increasing accuracies along the hierarchy (Figures 4A,B).

When averaging the results over networks, the individual identifiability of the four conditions was ordered as baseline < backward < scrambled < intact. This pattern was in line with the results obtained from the whole-brain connectome (Figure 4C). When averaging the results over conditions, the SR of networks was ordered as auditory network < language network < DMN < frontoparietal. Further analyses showed, compared with the other three networks, there was significantly lower between-subject similarity (or higher inter-subject variability) in the FCs profiles of the frontoparietal network (Figure 4D).

### The results of validations

#### Reproduce the findings with a different brain atlas

Applying the Schaefer atlas to assess the brain connectivities, we obtained a similar pattern of individual identifiability as the main analyses. The SR of identification increased along the language hierarchy: 56.17% for the baseline, 81.48% for the backward condition, 85.19% for the sentence-scrambled condition, and 87.04% for the intact condition.

#### Identification using a different strategy to pair conditions

The analyses using a different strategy to pair conditions for identification yielded a similar picture as the main analyses. The SR in identifying individuals between the rest and intact conditions was the lowest (70.37%), which increased to 84.26% between the backward and intact conditions. It then dropped slightly to 83.3% between the sentence-scrambled and the intact conditions and rose to 100% between the two sessions of the intact condition. We note that the overall SRs were higher than those of the main analyses, likely due to the use of more data points to compute the FCs.

## Discussion

Establishing the link between individual differences in the brain with the differences in cognition, behavior, and dysfunctions is a major goal of cognitive neuroscience. To fulfill this goal, the neuroscientific community, which has been mainly focused on the generic patterns of brain activities shared across the population, is now moving forward to characterize brain patterns that are robust and unique to individuals. Existing studies have discovered a set of brain features that can be used to distinguish individuals from each other and may serve as “brain fingerprints.” However, what is the information encoded in the brain that makes us unique remains elusive.

To understand the source of functional brain individualization, we explored the degree of individual identifiability along the language hierarchy. Subjects were scanned with fMRI during a resting state and when listening to backward-played, sentence-scrambled, and intact stories. For each task, the imaging data were collected from two scan sessions. Extracting the whole-brain FC profiles as features, we found that the individual identifiability tends to increase along the language hierarchy. The identification between the resting state and each of the task states achieved an average SR of 62.9% (the baseline). The mean SR across the two sessions for the backward condition (the low level) increased to 85.2%, which decreased slightly for the scrambled condition (83.3%) (the middle level), and then rose to 90.7% for the intact condition (the high level). This pattern was also observed when using the FCs of single networks (the language network and the frontoparietal network) to characterize individuals. In addition, we obtained a similar pattern by employing a different brain atlas to compute the brain connectome and by applying a different strategy of condition pairing for the identification.

### Increased individual identifiability along the language hierarchy

Using whole-brain FCs as features, we identified individuals across the resting and task sessions with an average SR greater than 60%. This performance is close to the results of previous studies which examined the individual identifiability across resting states and a set of tasks involving emotion, motor, memory, and language processing (Finn et al., 2015; Kaufmann et al., 2017). Together with previous studies, our work suggests that the state-independent, intrinsic processes are the major contributor to brain fingerprints.

Compared to the baseline, the SR in identifying subjects under the backward condition (the low-level) improved significantly by about 22%. One possibility is that this improvement reflected the *direct* contribution of low-order acoustic processing to brain fingerprints. However, when looking into single functional networks, the auditory network only obtained an identification accuracy of 44% for this condition, which was the worst among the four networks. Instead, it was the DMN that showed the highest SR (74%) in identifying individuals under the backward condition. These results seem to argue against the possibility of a direct contribution, suggesting that the low-order auditory perceptual process *per se* may not provide critical information in characterizing individuals. Alternatively, we propose that the presence of audio streams may constrain the activities of the auditory network as well as other brain networks (especially the DMN) as a whole, leading to a high degree of within-subject stability in brain connectivities across sessions (as can be seen in Figures 4A,C). This may explain the significant improvement in identifying individuals based on the whole-brain FCs.

Compared to the low-level condition, the SR in identifying individuals under the sentence-scrambled condition (the middle level) based on whole-brain FCs decreased slightly by about 2%. However, when taking the FCs of single functional networks as features, both the language network and the frontoparietal network performed better in identifying individuals under the scrambled condition than that under the backward condition. This pattern was also found in the averaged performance across the four networks. The increase in identifiability was accompanied by the increase in within-subject stability and a slight drop in inter-subject variability. These results suggest that, compared to listening to the meaningless backward-played story, comprehending sentences may help to blur irrelevant features (background noises), therefore enhancing those key individual features meanwhile making subjects’ FCs more similar to one another.

The greatest SR was obtained for the identification of individual subjects under the intact condition (the high level). Compared to the other two task conditions, there was greater inter-subject variability and slightly lower within-subject stability in brain FCs under the intact condition. The increased individual identifiability may be related to the fact that comprehending stories requires the integration of information over a longer time scale than did the two lower-level tasks. This is consistent with previous findings that the best identification emerges at longer time scales (Van De Ville et al., 2021).

### The differences among networks in distinguishing individuals

Among the four networks, the auditory networks consistently showed the lowest SR in discriminating subjects under all four conditions. Further analyses revealed that, while the within-subject FC similarity was the greatest, the inter-subject FC variability was the least in the auditory network among the four networks. The low inter-subject variability (or high inter-subject similarity) in the auditory network across the language hierarchy is consistent with previous findings (Lerner et al., 2011), which likely explains the comparatively poor performance of this network in distinguishing individuals.

The condition-averaged SR derived from the language network was higher than that derived from the auditory network but lower than that derived from the DMN. In comparison with the auditory network, both the language network and the DMN were characterized by greater inter-subject variability but lower within-subject stability.

The frontoparietal network consistently showed the best performance in identification under all conditions except for the backward condition. Under the intact condition, identification using the FCs of the frontoparietal network alone performed even better than that using the whole-brain FCs. Moreover, similar to the whole-brain FC profiles, the SR obtained using the FC profiles of the frontoparietal network also increased along the language hierarchy. Still, this increase was accompanied by the increase in inter-subject variability rather than within-subject stability of FCs. These findings are consistent with previous reports about a high degree of individualization (Finn et al., 2015; Amico and Goni, 2018; Horien et al., 2019) and inter-subject variability in the frontoparietal network (Mueller et al., 2013). Our study extends previous work by suggesting that the special role of the frontoparietal network in individual identification is likely owing to its function rather than its anatomical features.

### Limitations and implications

One limitation of the current study is the small sample of subjects and the short data length used to compute the FC. Despite that we have validated the main results with the analysis using a different data length (32 versus 64 time points), future studies based on a larger sample size are required to replicate our findings and evaluate the effect of data length (stimuli duration). Besides, all the subjects recruited in this study are females. Whether the results can be extended to males remains to be tested. Finally, while we observed that the more complex cognitive task and the network associated with higher-level cognitive functions tended to better distinguish individuals, most of the changes in identification accuracy did not reach statistical significance. Although this trend was reproducible across the analyses with different brain atlas and different strategies of condition pair, more work is needed to establish that this trend is meaningful rather than arbitrary.

Despite the above limitations, the current study may provide useful implications for future research. First, we demonstrated that, during the high-order story comprehension task, the brain functional connectivities are quite different across subjects but stable enough across time within the same subjects. Meanwhile, on the low-order perceptual task, the brain connectivities were quite stable across time and similar across individuals. These results imply that, for low-order functions, conclusions about the brain obtained from a relatively small pool of subjects can be generalized to larger groups. However, for high-order functions, averaged brain patterns obtained from a small sample may not well represent the general principles of brain function. However, if properly exploited, individual differences in brain activities on high-order tasks can provide useful information that is beyond what can be captured by those group-mean focused approaches (Liu et al., 2020).

Second, in line with previous work (Mueller et al., 2013; Finn et al., 2015; Amico and Goni, 2018), this study highlights the special role of the frontoparietal network in characterizing individuals. In addition, we found that the individual distinguishing ability of the frontoparietal network increased with the complexity of tasks. Based on these findings, we speculate that executive processes, which are the typical function of the frontoparietal network and demands for it usually increase with the complexity of tasks, might be the core factor underlying inter-individual differences in the brain and behavior. Future studies aiming to manipulate brain states to maximize individual differences may give priority to tasks involving executive processes.

### Conclusion

This study demonstrated that individual identifiability tended to increase along the language hierarchy: the more complex the task was, the better subjects were distinguished from each other based on their functional brain data. A similar principle was also found at the functional network level: compared to the low-order network (the auditory network), the high-order network was more individualized (the DMN and frontoparietal networks). Moreover, in both cases, the increase in individual identifiability was accompanied by the increase in inter-subject variability of the FC profiles. The two folds of results together suggest that, compared to the low-order functions, the high-order functions of the brain are more important in making us unique. At the same time, task-independent neural processes seem to contribute more than task-evoked neural processes to brain individualization. What is exactly encoded in the task-independent brain activities and its function in cognition and behavior remain an open question.

## Data availability statement

The data that supports the findings of this study are available from the corresponding authors, upon reasonable request.

## Ethics statement

The studies involving human participants were reviewed and approved by Beijing Normal University. The patients/participants provided their written informed consent to participate in this study.

## Author contributions

JZ: conceptualization, investigation, methodology, and writing. LZ: conceptualization, methodology, and writing. JJ: writing—review and editing. MY, SL, and XT: investigation and project administration. YM: writing—review and editing. LL and GD: conceptualization, methodology, writing—review and editing, and supervision. All authors contributed to the article and approved the submitted version.

## Footnotes

1
http://rfmri.org/dpabi

2
http://www.fil.ion.ucl.ac.uk/spm

3
https://www.neurosynth.org
