# Supplementary material for: Brain fingerprints along the language hierarchy
Source: Front Hum Neurosci. 2022 Sep 15;16:982905. doi: 10.3389/fnhum.2022.982905 (PMC9521489; doi:10.3389/fnhum.2022.982905)
Supplement: Supplementary file 1 [file Data_Sheet_1.docx]

# Brain fingerprints along the language hierarchy

Supplementary material


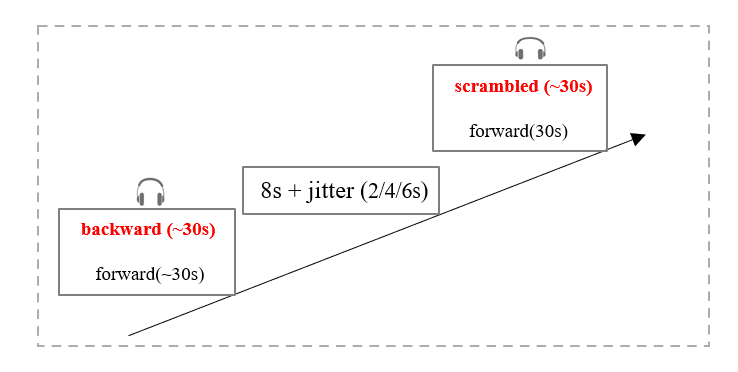


**Figure S1.** Experimental protocol for the backward and sentence-scrambled conditions. Experimental stimuli were presented in four blocks (two for each condition) within an fMRI scan session. Each block lasted 60-62s (30-31 TRs), during which a story with the first half played backward (or sentence scrambled) and the second half kept intact was presented. For each condition, only the first half of time series in a block were extracted and the data from two blocks were concatenated, resulting in a total of 32 time points used for computing functional connectivities. For each subject, two such scan sessions were acquired.

**Figure S2.** Experimental protocol for the intact condition.
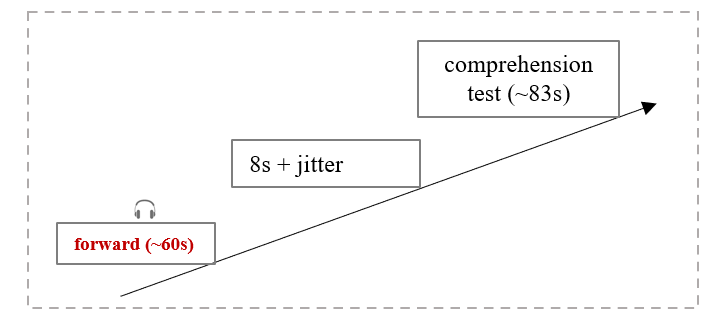
 Within an fMRI scan session, four intact stories were played, each lasting about 60s. After listening to a story, the subject performed a comprehension test. To keep the data length consistent with the other two conditions, we only extracted the time series of the first story to compute brain functional connectivities. For each subject, two such scan sessions were acquired.

## Functional network definition

For each participant, an FC matrix was constructed based on the Shen-368 atlas. The FC matrices from all participants were then averaged. Community detection was performed on the group-averaged FC matrix applying the Louvain clustering algorithm in the Brain connectivity toolbox (<https://sites.google.com/site/bctnet/>). To determine the Gamma parameter in the algorithm, we took the approach used by Ji (Ji et al. 2019). The criteria included: i): separation of primary sensory-motor network (visual, auditory and somatomotor) from all other networks. ii): high similarity of network partitions across nearby parameters (stability). iii): high with-network connectivity relative to between-network connectivity (high modularity).

A set of gamma values ranging from 1.2 to 2.5 were tested. For every tested gamma, we ran the algorithm 1,000 times and measured how consistent a given partition was to every other partition using the z-rand score. Each z-rand score averaged across the iterations was then multiplied by its corresponding modularity score to find a modularity-weighted z-rand score. Finally, the gamma value (gamma =2.5) corresponding to the peak of the modularity-weighted z-rand score meanwhile satisfied the criteria of finding a plausible number of networks including primary sensory/motor networks was selected.

We implemented network partition using codes adapted from a prior study (Barnett et al. 2021).

**Reference**

Barnett AJ, Reilly W, Dimsdale-Zucker HR, Mizrak E, Reagh Z, Ranganath C. 2021. Intrinsic connectivity reveals functionally distinct cortico-hippocampal networks in the human brain. PLoS Biol. 19:e3001275.

Ji JL, Spronk M, Kulkarni K, Repovš G, Anticevic A, Cole MW. 2019. Mapping the human brain's cortical-subcortical functional network organization. NeuroImage. 185:35-57.
